# Cure or Carcinogen? A Framing Analysis of European Radon Spa Websites

**DOI:** 10.3389/ijph.2022.1604559

**Published:** 2022-04-22

**Authors:** Robbe Geysmans, Tanja Perko, Mirjana Keser, Christiane Pölzl-Viol, Ivana Fojtíková, Peter Mihók

**Affiliations:** ^1^ Belgian Nuclear Research Centre, Mol, Belgium; ^2^ Austrian Agency for Health and Food Safety (AGES), Vienna, Austria; ^3^ Federal Office for Radiation Protection, Oberschleissheim-Neuherberg, Germany; ^4^ National Radiation Protection Institute, Prague, Czechia; ^5^ Research and Innovation Centre, Faculty of Economics, Matej Bel University, Banská Bystrica, Slovakia

**Keywords:** public health, health communication, radon, frame analysis, spas, radon spa

## Abstract

**Objectives:** Radon, a radioactive gas, is among the leading causes of lung-cancer worldwide. While public health authorities emphasize radon’s health risks, there are spas across Europe which claim health benefits of radon. This study investigates how websites of European radon spas frame radon gas, in order to understand the potential controversy between “radon as carcinogen” and “radon as cure,” and its potential impact on public health interventions.

**Methods:** A two-phased frame analysis of websites of radon spas (*n* = 26) situated in the European Union.

**Results:** Five frames are identified, which present radon as a 1) source of health, 2) natural gas, 3) (non) risk, 4) luxury and 5) fountain of youth. These five partly overlapping frames are at times in clear contrast with the ways in which radon is presented in a public health context.

**Conclusion:** Being aware of the existence and contents of radon frames, which potentially challenge or contradict public health interventions, helps responsible authorities in designing more effective campaigns and interventions.

## Introduction

Radon, a colorless and odorless gas, has been described by the World Health Organization as one of the main sources of exposure to natural ionizing radiation [1]. Epidemiological evidence has shown how radon increases the risk of lung cancer, for example among underground miners, but also among the general public, who might be exposed to elevated radon levels in their homes [2]. Since the 1980s, radon has therefore been treated as a carcinogen, and is generally considered as one of the leading causes of lung cancer worldwide [3, 4]. Over the years, health authorities at local, national, inter- and supra-national levels have invested in informing the public about and protecting it from the risks of this radioactive gas. In Europe, for example, the 2013 directive on “basic safety standards for protection against the dangers arising from exposure to ionising radiation” contains articles both on limiting indoor radon exposure in residential buildings and in the workplace. This directive requires that EU member states establish a radon action plan in order to address the long-term risks from radon exposures for any source of radon ingress, whether from soil, building materials or water [5].

Overall, the consensus thus seems to be that radon is a potential public health hazard. Given the dominant understanding of radon as a threat to public health, it is remarkable that across the world there are facilities which offer services based on claimed health benefits of radon gas. In such facilities, people can, for example, bathe in radon rich water or breathe radon rich air (e.g., in former mining tunnels). In this paper, we will refer to these facilities with the generic term “radon spas” [6, 7]. In the European Union, radon spas can be found in at least nine different countries. The therapies and treatments at these spas can in some cases be traced back to the medieval ages [8, 9], and as such sometimes substantially predate the “discovery” of radon by Rutherford and Owens in 1899. Despite (or because of) this substantial history, the topic of health benefits attributed to radon is highly controversial, as modern medicine has certainly not unequivocally embraced the use of radon in medical treatments [7].

Interestingly, researchers have taken a significant interest in studying communication efforts and campaigns organized by authorities, which focus on the health risks of radon [10–14]. Radon spas and their communication materials, however, have remained highly underexplored (for an exception, see the works of Barbara Erickson or a self-published book of Dietrich Liechti [15–18]). Nevertheless, they are a peculiar and potentially relevant actor in shaping people’s understandings of and ensuing actions with regard to radon gas.

In this article, we are not taking any position in the controversy regarding the health effects of radon. It is not our objective to assess health claims, nor are we interested in actual radon levels in the studied radon spas. What we will focus upon, however, is the ways in which these spas communicate about radon to the general public. We believe this communication is relevant, as it offers a certain framing which might be different from the dominant message on radon communicated by (inter-)national authorities, which emphasize the threat posed by radon. Communication is not without consequences: a message selects, highlights, and/or hides certain features of what happens or is important, and as such provides a specific representation of reality—a frame through which we make sense of our world [19, 20].

We investigate the research question “how radon spas frame radon gas on their public websites.” We focused on radon spas located in the European Union and conducted a frame analysis of the webpages of 26 of these spas. We argue that the way radon spas communicate about radon online, reveals the promotion of (a) reality/realities in which radon has particular characteristics and effects, which differ from the characteristics and effects highlighted in public health campaigns. This entails, among others, an added complexity regarding the design and implementation of said health campaigns, especially in those countries where radon spas are present. It has repeatedly been highlighted how “the more support a campaign has from other sources, the easier it will be to get a new message disseminated,” while “opposition to [a] campaign message position makes the task more difficult” [21, 22].

In the next section a conceptual introduction to the notion of framing is provided. After that, we present the two-staged methodology which was used to identify and analyze the radon frames. These identified frames are discussed in section four, before the paper concludes with a reflection on implications of and future research regarding radon spas in a public health context.

### Theoretical Background: Framing Radon

The concepts of frame and frame analysis can be traced back to the 1950s, and gained popularity in sociology through the 1974 seminal work “Frame analysis: An essay on the organization of experience” by Ervin Goffman. In this book, Goffman presents and discusses some of the basic frameworks people use for making sense of society. The underlying assumption here is that “definitions of a situation are built up in accordance with principles of organization which govern events -at least social ones- and our subjective involvement in them” [23]. It is especially since the end of the 1980s that the interest in frames and frame analysis has soared, particularly in the fields of social movement research and communication studies. It is from the latter field that the conceptual underpinnings of this article are mostly drawn.

In media analysis it is now commonplace to highlight how news reporting (in television, newspapers, the internet etc.) uses frames which guide and influence the ways the public understands events and phenomena [19, 24, 25]. Given this widespread interest in frames and framing, an equally impressive number of definitions has been presented to describe the concept of framing [24, 26, 27]. One of the most commonly used definitions comes from Entman, who states that “to frame is to select some aspects of a perceived reality and make them more salient in a communicating text, in such a way as to promote a particular problem definition, causal interpretation, moral evaluation, and/or treatment recommendation for the item described” [19].

Particularly relevant in this context, is that frames can be multiple and conflicting, as they are part of broader interpretive struggles between (potentially unequal) actors, which shape meanings according to their worldviews and agendas [28]. Analyzing the US media discourse on nuclear power between 1945 and the 1980s, Gamson and Modigliani, for example, identify seven different frames which are used in the media [24]. Some of these are clearly conflicting on certain aspects, and competing for attention, making them more dominant in one particular era (e.g., a framing of nuclear power focused on “progress” at the dawn of the nuclear age), while being challenged in other eras (e.g., by frames which stress the safety issues related to radiation and nuclear power from the 1970s onwards). In the case of radon gas, it is thus quite possible that frames which stress the threats posed by radon exposure, are co-existent with frames which stress its benefits.

Key in identifying frames, is being attentive to the framing devices which can be found in specific communication materials (e.g., a newspaper article, a television program, a website or a cartoon). Framing devices characterize a frame, and are key in understanding what aspects of a topic are emphasized, downplayed, or omitted through a communication source. Such framing devices can be metaphors (e.g., radon as a “silent killer”), catchphrases (e.g., test your house!), visual images (e.g., radiation warning symbols), etc. [19, 24].

We assume that radon spas have a particular way of framing radon gas, and it is through the content of their communication that we get insight into these particularities (and how these might differ from public authorities’ framing focused on radon as a threat to health). As a particular communication source, we focus on the websites of radon spas (see the next section for an elaboration).

The central research question guiding this study is therefore which radon frames can be encountered on the websites of radon spas situated in the European Union.

## Methods

When focusing on radon spas, a main source of communication are the spas’ own websites, on which their services are presented to the broader public. It is these websites on which our analysis will build. The identification of radon spa websites happened through a combination of search strategies. An existing list of facilities was obtained from the European Association of Radon Spas EURADON. This was extended with an elaborate internet search, combining both classical search engines (i.e., Google) and the search function of a popular travel website (tripadvisor.com). On this travel website, reviews of facilities and activities were searched for the word “radon,” which generated over 120 hits. Finally, we consulted different partners in the Horizon2020 RadoNorm project to enquire whether they had knowledge of any radon facility in their respective countries. After checking the identified spas’ locations (only spas in the EU were included), services (to be included, they needed to offer one or more services linked to radon) and website language (only websites available in English and/or German were included), we obtained a final list of 26 websites.

These websites were studied in two interconnected phases. In the inductive phase, a hermeneutic approach was used to identify radon frames [29]. This was followed by a second, deductive phase, in which another set of researchers checked the found frames against an extended dataset of websites. In both phases, multiple coders were involved, in order to minimize the influence of researchers’ own mental constructs on the identification of a frame [30].

In the inductive phase, 17 radon spa websites were used for the first, bottom-up identification of frames, focusing on websites which had an English version available. A double-coder strategy was used in order to minimize the arbitrariness and subjectivity often connected to inductive hermeneutic frame analysis [31, 32]. Two research assistants carefully and independently read selected websites, taking into account both textual and visual elements. Particular attention was directed at how the websites defined or described radon and the radon-related services offered by the spa. The coders were asked to identify references to or depictions of radon gas, treatments, target audiences, effects (benefits or risks), and the broader facility and its surroundings. Based on the combined findings of both coders, the principal investigators identified five frames through which radon is presented on the analyzed websites.

In the deductive phase, these identified frames were put to the test, by having a second set of coders check for their prevalence on an extended list of websites (*N* = 26), also containing webpages in German. A training was provided to the coders, in order to familiarize them with the five frames. Key questions were provided for each frame, facilitating the process of identification. For the framing of radon as a healing source, these questions, for example, were “is there any mentioning of health benefits of radon?”, “is there any mentioning of diseases or conditions which can be treated?” “Are there any depictions of health/medical related persons, objects, or other visuals (e.g. doctor’s coats, epicurean symbols)?” This second round of analysis confirmed the five frames identified.

It should be noted that between the first, inductive phase of the analysis, and the second phase, some websites had been updated and adapted. In one case, the English version of a website had even entirely gone offline, meaning it was included in phase 1, but not anymore in the final analysis (Table 1).

**TABLE 1:** Analyzed radon spa websites per European Union member state (2021).

Phase 1 (*N* = 17)		Phase 2 (*N* = 26)	
Country	Number of radon spas	Country	Number of radon spas
Austria	6	Austria	7
Bulgaria	1	Bulgaria	2
Croatia	1	Croatia	1
Czech Republic	1	Czech Republic	1
Germany	5	Germany	9
Greece	1	Greece	1
Hungary	0	Hungary	1
Italy	1	Italy	2
Poland	1	Poland	2

In the next section, each identified frame will be illustrated and discussed. When presenting quotes from websites, spa names are not mentioned, but a letter (A–Z) will be used, where each letter stands for one of the spas in our dataset. Also, German quotes were translated to English.

## Results

A total of five different but partly overlapping radon frames were identified. These five frames present radon respectively as 1) a source of health, 2) a natural gas, 3) a (non) risk, 4) a luxury and 5) a fountain of youth. Below, each of these frames are discussed separately, in the order of the number of websites on which they were identified.

### Radon as a Source of Health

On all websites which were analyzed, radon was framed as a source of health, meaning that it was presented as something which would alleviate or even cure several diseases and health-related discomforts. By being exposed to radon, visitors could, for example, benefit from its “effective pain relief and anti-inflammation” (Spa B) or its claimed positive influence on “disorders of the locomotor system and asthma” (Spa L), to name only a few of the medicinal powers attributed to radon gas by the analyzed websites. This frame is supported by various visuals, depicting what seem to be medical settings, in which spa visitors are consulting or being treated by people wearing doctors’ coats or nurses’ uniforms.

To legitimize the idea of radon gas as a source of health, this frame builds on two strategies. First, it connects radon’s claimed health benefits to science. Here, legitimacy is drawn from references to scientific proof provided by experiments, double-blind studies, or significant numbers of scientific publications. Visitors to the website of radon spa Y can, for example, learn how “various studies indicate a pain-relieving and anti-inflammatory effect of radon,” and spa A states how through “precisely controlled double-blind studies, in which neither the patients nor the examining doctors knew which patient received radon and which did not, its therapeutic effectiveness was substantiated.” These framing devices emphasize empiricism and rationality, and as such help communicate the notion of radon as a legitimate medicine. These calls to empiricism and scientific rationality are in many ways similar to the historical ways in which modern medicine gained legitimacy [33]. A second strategy for legitimizing radon as a healing source, is through emphasizing the long periods of time over which the gas has been attributed with/used because of its healing powers. By emphasizing how radon “has been valued as a remedy for over a century” (Spa X) and how “local miners knew this in 1900, when they healed their wounds by immersing them in this “magical” water” (Spa R), legitimation is sought in historical narrative and tradition.

### Radon as a Natural Gas

A second frame, found on 25 out of 26 websites, presents radon as a natural gas, by emphasizing its natural origins and characteristics. Visually, this frame is supported by pictures of wide landscapes, mountains and rocks, caves and mines, and lakes, springs or rivers. Radon is often described as a natural remedy or a treasure of nature, and emphasis is put on its presence in “natural springs” (e.g., Spa K) or in “air and earth” (Spa Y). Its origins are described as laying in “the entrails of the earth” (Spa M), or—more elaborately—as the consequence of “a series of successful geological processes over a considerable period of several million years” (Spa E).

### Radon as a (Non) Risk

A third frame relates radon to the presence and/or absence of risk. On 16 out of 26 websites, radon and/or radon therapies are framed as risky or containing a certain need for precaution. The website of Spa C, for example, reads how “radioactive radiation in high doses can cause cancer or harm unborn babies in the womb,” and also Spa W mentions “the fact that high doses pose an undisputed risk of lung cancer.” However, references to (lung) cancer are rather rare (only being mentioned on three websites), and most websites frame risk in much more implicit terms. In these more implicit framings, the risk of radon is brought forward by mentioning that therapies can only be taken after a doctor’s visit, or by excluding certain groups (e.g., pregnant women) from radon therapy. Why this doctor’s visit is necessary or why these groups cannot be exposed to radon is not explained. As such, what the risk exactly entails remains unclear. Visually, the framing of radon as a risk is not supported (except on one website which warns pregnant women that they cannot take radon therapy).

Moreover, on 9 out of 16 websites which frame radon as risky, a counter-frame is also presented which minimizes the risk, or reassures the reader that there is no need to worry. In addition, two websites provide only such reassurance, without a reference to potential risk as such. In some cases, such reassurance is provided through reference to a form of external control or oversight. Spa B, for example, states that it “has all the necessary radiation protection permits” and Spa M highlights how “as part of the regular measurement of air by [the national nuclear regulatory authority] it was found that […] one person could pass more than 800 baths per year to reach the maximum limit of inhaled radon in these areas.” In other cases, the reader more explicitly has to trust on the claims and expertise of the spa and its staff. Statements like “radon treatment has no side effects,” “the best cure taken in excess is harmful and dangerous poison applied in minimum amounts becomes the cure” (Spa L) or “radon therapy […] is naturally gentle and without known side effects” (Spa U) are telling in this sense.

### Radon as a Luxury

A fourth frame presents radon gas as a luxury, and was encountered on 21 out of 26 websites. The framing devices coded under this frame refer to radon as an exclusive substance, with unique and desirable characteristics or a rare prevalence. As such, being able to be exposed to and benefit from radon is a luxury, and visiting a radon spa makes this splendor accessible and affordable. Radon provides a sort of unique selling proposition to the radon spa: on the analyzed websites, links are regularly drawn between the spa, its location and its access to/use of radon in order to highlight its uniqueness and desirability, thus setting it apart from potential competitors. The exclusive character of radon is emphasized by quotes such as “the rare noble gas radon is one of the most effective remedies in spa science” (Spa C), “the ionisation, the mineral salt content, and the special gases and metals also make the water so distinct” (Spa P) or “the unique combination of the precious and rare noble gas radon and the cold chamber, as a non-drug form of pain therapy, are proving to be our recipe for success” (Spa F).

Furthermore, radon is often referred to as a natural noble gas on the analyzed websites. Obviously, the “noble” in this context can be interpreted as referring to is classification as a chemical element. Radon is one of seven noble gases listed in the periodic table, which all share similar properties, e.g., a very low chemical reactivity. The sheer multitude of references to the “noble” gas is, however, striking. This multiple mentioning of “noble” hints at another meaning of the word: something which is “grand,” “majestic” or of high quality, hence implicitly strengthening the framing of radon as a luxury.

Finally, a visit to the radon spa is also presented by some analyzed websites as an indulgence because of the relaxing, luxurious and comfortable atmosphere offered. Spa H, for example, seduces customers with the catchword “let us pamper you” and Spa L quite lyrically targets potential customers by stating that “in a specific atmosphere of a picturesque adit, lying comfortably, being protected against excessive chill and listening to relaxing music you have an opportunity to breathe in cold and humid air.” In these instances, an implicit link is made between radon gas and the treat of visiting a lush spa, thus also reinforcing the framing of radon gas as a unique luxury, an exclusivity. This frame is further supported by numerous visuals of inviting spa environments with marble interiors, tempting pools and happy people indulging in different spa treatments.

### Radon as a Fountain of Youth

Finally, on half of the websites, radon gas is framed as a fountain of youth. This fifth frame on first sight is closely related to the first frame in which radon is presented as a healing source. Indeed, here as well reference is made to the beneficial powers of radon, but this time emphasis is not so much on healing, but rather—arguably taking this a step further—on rejuvenation. Radon makes you young (again), it revitalizes your body and provides strength. Websites talk about “regaining vitality” (Spa D), the “radon fountain of youth” (Spa F), a “rejuvenating effect” (Spa I), or how “radon accelerates the renewal processes in tissues” (Spa K).

Visually, this frame is supported by numerous pictures of young people taking baths and other radon therapies. This use of young people in visual materials is particularly noteworthy because we can assume that these are not the prime clientele of the spas. In a study on visitors of a radon health mine in Montana (United States), Erickson noted how 84% of the more than 800 visitors she analyzed were aged 60 years or older [16].

## Discussion

Social scientific research has taken a significant interest in studying public authorities’ radon communication efforts and campaigns [10–13]. In such public health campaigns, a dominant way of framing radon is in terms of risks and hazards. For example, a recent content analysis of 173 public authority websites in eight European member states found, among others, the following messages: “take the test to protect yourself”, “radon: an enemy in your house”; “the radon risk”; “Test. Fix. Save a Life” [12].

At the same time, in at least nine European member states, spas can be found which provide services based on claimed health benefits of radon gas. These radon spas have, however, largely remained under the radar of social science, despite the fact that they might provide alternative or even contradictory frames on radon, and hence can potentially impact radon communication campaigns. Having analyzed 26 websites of European radon spas, this article shows how these websites make information on radon available to the public and communicate about radon as a cure (instead of a carcinogen) in a consistent and engaging way. More specifically, five distinct radon frames were identified, which present radon as 1) a source of health, 2) a natural gas, 3) a (non) risk, 4) a luxury and 5) a fountain of youth. Comparing these frames with how public authorities tend to communicate on risk, feeds the impression that we are in fact dealing with two different substances. While radon spas frame radon as a source of health, health campaigns try to convey the message that radon “is an important public health issue that requires action” [3]. Furthermore, the extensive attention spa websites put on radon’s natural origins and characteristics stands in contrast to recent advice formulated for those designing health campaigns. This latter advice builds on the notion that people “perceive technological threats to be more risky than natural threats” [34], and hence stresses that “risk communicators need to draw attention to radon” not as a natural gas, but rather as a major cause of “indoor air pollution” [35]. Thirdly, radon spa websites mostly provide implicit and non-visual references to radon risk (if any), while health campaigns contain numerous explicit references to the lung cancer risk associated with radon, often supported by various visuals (see e.g., the websites of the 2021 US radon awareness week https://www.cdc.gov/radon/awareness.html, the 2021 European radon week https://radoneurope.org/event/european-radon-week-2021/, or the 2020 UK radon awareness week https://radonweek.co.uk/). And fourth, spa websites present radon as a rare and exceptional element, which can be encountered at unique locations, while health communication stresses the abundance of radon by emphasizing that “it can enter any building: homes, offices, schools” [36]. Highlighting the existence of these different frames demonstrates that radon communication can be controversial.

Moreover, while indeed the impression might arise that we are dealing with two entirely different radon realities, these realities share social and physical spaces, affecting overlapping audiences. This paper hence provides a first step towards recognizing that different and sometimes contradicting frames on radon exist, and offers a plea to recognize the potential role of radon spas in shaping people’s awareness, perceptions and actions with regard to radon gas. There are indications that some public authorities already demonstrated such recognition in legal frameworks for radiation protection. The German radon action plan, for example, states how a measurement obligation exists for specific workplaces, explicitly listing radon baths as a place where workers need to be protected from radon. Similarly, public health campaigns need to at least be aware of these spas, the ways in which they frame radon, and the potential effects these alternative framings might have on their audiences. One of these effects might be that citizens perceive the threat of radon as insignificant, hence making it less likely that they act upon the issue by testing and/or remediating their homes [35]. The 2013 BSS Directive stipulates that all EU member states are legally required to design a public communication strategy to increase awareness on radon risk, and hence decrease lung cancer rates due to radon in dwellings, public buildings and at work [5]. We argue that especially in regions were radon spas are present, a successful public communication strategy would require a reflection on how to deal with the radon communication offered by these actors.

This article offers a first attempt at understanding radon spas in the context of radon as a public health issue. While framing can be an important way in which these spas impact perceptions and behaviors with regard to radon gas, future research should build on these insights to gain knowledge on the perceptions, attitudes and actions of these spas’ stakeholders, ranging from visitors, to employees and broader publics.
